# Systematic review of multidrug-resistant *Klebsiella pneumoniae* in the Arabian Peninsula: molecular epidemiology and resistance patterns

**DOI:** 10.3389/fmicb.2025.1489317

**Published:** 2025-01-24

**Authors:** Enaam K. Idrees, Marwh G. Aldriwesh, Manal M. Alkhulaifi, Majed F. Alghoribi

**Affiliations:** ^1^Department of Botany and Microbiology, College of Science, King Saud University, Riyadh, Saudi Arabia; ^2^Infectious Disease Research Department, King Abdullah International Medical Research Center, Riyadh, Saudi Arabia; ^3^Department of Clinical Laboratory Sciences, College of Applied Medical Sciences, King Saud bin Abdulaziz University for Health Sciences, Riyadh, Saudi Arabia; ^4^Ministry of the National Guard - Health Affairs, Riyadh, Saudi Arabia; ^5^Department of Basic Science, College of Science and Health Professions, King Saud Bin Abdulaziz University for Health Sciences, Riyadh, Saudi Arabia

**Keywords:** MDR *Klebsiella pneumoniae*, molecular epidemiology, antimicrobial resistance gene, carbapenem-resistant *Klebsiella pneumoniae*, the GHC countries, Arabian Peninsula

## Abstract

**Background:**

The rapid emergence of multidrug-resistant *Klebsiella pneumoniae* (MDR *K. pneumoniae*) is a major public health and economic burden worldwide. Various resistance mechanisms complicate treatment, leading to increased morbidity and mortality. Despite numerous studies conducted in Gulf Health Council (GHC) countries, the molecular epidemiology of MDR *K. pneumoniae* remains not clearly defined. This systematic review aims to analyze the emergence of antimicrobial resistance genes in MDR *K. pneumoniae* across GHC countries.

**Methods:**

A systematic search was conducted using PubMed, ScienceDirect, and OpenMD for articles published up to March 15, 2023. The search strategy focused on the bacterial name, drug-resistance genotypes, and GHC countries. The review followed PRISMA guidelines, with two independent reviewers assessing the risk of bias using NIH Study Quality Assessment tools.

**Results:**

The primary search yielded 1,663 studies, of which 67 met the inclusion criteria. Saudi Arabia contributed the most studies, with 41 (61.1%), followed by Kuwait with 7 (10.4%), and the UAE with 6 (9%) studies. Oman and Qatar each contributed 4 studies (6%), and Bahrain contributed three studies (4.5%). The remaining 4 studies (4.4%) were from multiple GHC countries. The studies exhibited considerable heterogeneity in detection methods, target genes, and resistance mechanisms. Notably, only one environmental study was conducted in the UAE, and one community-based study in Kuwait, while the remaining studies focused on clinical samples. Various resistance mechanisms and patterns were observed between countries and across different years within the same country. The review highlighted the widespread prevalence of ESBL genes, particularly *bla*_TEM_ and *bla*_CTX-M-15_, and the emergence of carbapenemase genes such as *bla*_OXA-48_ and *bla*_NDM-1_ and *bla*_KPC-2_. Additionally, colistin resistance through the *mcr-1* gene and *mgrB* mutations was reported in Saudi Arabia and the UAE, posing a significant public health challenge.

**Conclusion:**

Data from GHC countries shows significant gaps, particularly in community and environmental and molecular epidemiology studies. Limited molecular and genome-based investigations hinder comprehensive AMR surveillance. Implementing standardized methodologies and fostering molecular and genome-based AMR surveillance programs at both national and regional levels within the GHC are essential for effectively combating the spread of MDR *K. pneumoniae* and improving public health outcomes in the region.

## 1 Introduction

In modern medicine, the emergence of *Klebsiella pneumoniae* strains exhibiting multidrug resistance (MDR), extensive drug resistance (XDR), and pandrug resistance (PDR), as well as the production of extended-spectrum β-lactamases (ESBL) and/or carbapenemases, represents a growing global crisis that urgently calls for the development of new antibiotics. MDR refers to bacterial strains resistant to at least one agent in three or more antimicrobial categories, XDR denotes resistance to all but one or two antimicrobial categories, and PDR signifies resistance to all agents in all antimicrobial categories, leaving no effective treatments (Magiorakos et al., 2012; Shamsuzzaman, 2015). In response to this ever-increasing threat, the World Health Organization (WHO) recently released the 2024 Bacterial Priority Pathogens List (BPPL), categorizing these pathogens into priority groups to guide research and strategies for controlling antimicrobial resistance (WHO Bacterial Priority Pathogens List, 2024). These pathogens are listed as a significant threat to public health as they can cause severe and life-threatening infections such as bloodstream infections and pneumonia. Available treatment options, including last-resort antibiotics, have become limited and ineffective due to the acquired resistance mechanisms (Hersh et al., 2012; Petrosillo et al., 2019; Rodríguez-Santiago et al., 2021; Wang et al., 2020; Pu et al., 2023; Aris et al., 2020; Cain et al., 2018).

In both humans and animals, the gastrointestinal system and oropharynx are naturally colonized by the Gram-negative bacterium *K. pneumoniae*. However, due to its capacity to cause community-acquired illnesses such as necrotizing pneumonia, liver abscesses, and endogenous endophthalmitis, *K. pneumoniae* is considered the most clinically significant species within the *Klebsiella* genus (Podschun and Ullmann, 1998). Additionally, *K. pneumoniae* contributes to hospital-acquired severe infections, such as sepsis, surgical site infections, and urinary tract infections (Yong et al., 2009). *K. pneumoniae* has acquired and disseminated multiple MDR genes, including ESBL variants, carbapenemase genes, and colistin-resistance genes (Al-Zahrani and Alsiri, 2018; Navon-Venezia et al., 2017). Consequently, the emergence of MDR *K. pneumoniae* poses significant public health challenges, complicating treatment regimens and leading to increased morbidity and mortality rates. Common antibiotics, such as third-generation cephalosporins, aminoglycosides, fluoroquinolones, and carbapenems, continuously lose their effectiveness against *K. pneumoniae* (Rolain et al., 2010). The rise in carbapenem resistance in *K. pneumoniae* is a global issue associated with higher morbidity and mortality rates, as well as increased medical expenditures in Saudi Arabia and other neighboring countries (Poirel et al., 2011; Al-Abdely et al., 2021; Alraddadi et al., 2022; Sonnevend et al., 2022; Abid et al., 2021). The Gulf Health Council (GHC) countries (Saudi Arabia, United Arab Emirates, Kuwait, Qatar, Oman, and Bahrain) are not immune to this problem. Rapid urbanization, high healthcare utilization, and extensive international travel in these regions contribute to the spread of resistant strains (Weber et al., 2017; Aiesh et al., 2023; Yu et al., 2021; Berndtson, 2020; Bokhary et al., 2021; Frost et al., 2019). Moreover, the setting of mass gatherings, such as the annual Muslim pilgrimage, Hajj and Umrah, that take place in Saudi Arabia plays an essential role in the spread of diverse antimicrobial-resistant strains (Al-Tawfiq and Memish, 2021; Leangapichart et al., 2017; Setiawaty et al., 2022; Leangapichart et al., 2016).

In the past decade, advanced molecular techniques have been employed to identify antimicrobial resistance genes and track their dissemination, providing insight into genetic mechanisms and the transmission dynamics underlying antibiotic resistance in clinically significant pathogens (Salawudeen et al., 2023; WHO, 2020). Moreover, molecular epidemiology is crucial for understanding the clonal relationships among different *K. pneumoniae* isolates, providing essential insights for devising effective infection control strategies and guiding appropriate antibiotic regimes (Cireșă et al., 2024; Alghoribi et al., 2018).

Recognizing the gap in knowledge regarding the molecular epidemiology of antimicrobial resistance mechanisms in the Arabian Peninsula, the 2018 position paper by Alghoribi and colleagues called for genomic epidemiology to combat AMR (Alghoribi et al., 2018). They emphasized the importance of molecular investigation of AMR as a crucial tool for identifying emerging pathogens and their resistance mechanisms. In alignment with this call, the WHO’s technical note “GLASS Whole-Genome Sequencing (WGS) for Surveillance of Antimicrobial Resistance” (2020) outlines the benefits and limitations of molecular investigation for AMR surveillance (WHO, 2020). Although significant progress has been made, comprehensive information about the molecular epidemiology of *K. pneumoniae* and the prevalence of its resistance genes in the GHC countries remains limited.

A comprehensive understanding of the regional epidemiological patterns and resistance mechanisms is essential for addressing the public health threat posed by this pathogen. This systematic research aims to determine the status of molecular epidemiology and the genetic distribution of MDR *K. pneumoniae* in the GHC countries by highlighting the prevalence of crucial resistance genes and the distribution of dominant clones. By collating and analyzing data from various studies, this review will provide a detailed overview of the current status and identify gaps in the existing literature, thereby offering a foundation for future research and intervention efforts. Understanding the regional dynamics of MDR *K. pneumoniae* is imperative for developing targeted strategies to curb the spread of resistance and improve patient outcomes in the GHC countries.

## 2 Materials and methods

While systematic reviews have extensively reported the molecular epidemiology of MDR *K. pneumoniae*, including ESBL, carbapenemase-producing and colistin-resistant strains globally, there is a notable lack of evidence specific to GHC countries, underscoring the need for region-specific data to understand antimicrobial resistance dynamics. The guidelines of the Preferred Reporting Items for Systematic Reviews and Meta-Analyses (PRISMA)^1^ were followed in developing the current systematic review protocol using the PRISMA 2020 checklist (Page et al., 2021). When planning a search strategy, the PICO (Population, Intervention, Comparison, and Outcome) tool was used to list terms and keywords by the main concepts in the search question as an organizing framework accessed on Oct 20, 2023.^2^ A comprehensive systematic review was performed using the major electronic databases of PubMed, ScienceDirect, and OpenMD for articles published from inception to March 15, 2023. Consistent keywords and search strategies were applied across these databases containing the terms (molecular epidemiology, antimicrobial resistance genotypes, AMR genotypes, genetic diversity, clones, genotyping, antibiotic resistance genes, genetic analysis, resistome, whole genome sequencing OR genomic characterization) and (*Klebsiella pneumoniae*) and (Gulf Cooperation Council region). Moreover, two independent reviewers selected relevant studies from the references of found studies after removing the duplicates.

### 2.1 Eligibility criteria

The review included accessible full-text original articles published in English before March 15, 2023, without any restrictions on the publication year of the included studies. In addition, the systematic review included clinical studies, case reports, environmental and one-health approach studies that investigated the antimicrobial resistance genes in *K. pneumoniae* in the GHC countries using phenotypic and genotypic methods. All studies that addressed MDR *K. pneumoniae*, including ESBL, carbapenemase-producing and colistin-resistant strains, were included as a sub-population of *Enterobacterales*. In contrast, all studies reporting antimicrobial resistance mechanisms in *K. pneumoniae* using phenotypic detection methods were excluded from this review. Additional exclusion criteria included reviews, conference abstracts, study protocols, and studies performed outside the GHC countries.

### 2.2 Selection and data extraction

All references of extracted studies were imported to EndNote (version 21.2), where duplicates were removed. Two stages of screening were performed by two independent reviewers. The first screening stage included title and abstract screening of the imported references. Afterwards, all studies that were included during the first stage of screening underwent full-text screening during the second stage. The decision of each reviewer was taken blindly, and disagreements between researchers were resolved through discussion with a third reviewer. Following the two screening stages, all articles included were carried out for data extraction based on a data collection form designed to address the aim of the current review (Supplementary Table S1) using a Microsoft Excel worksheet. The data extracted from each study included the following: the citation, the country where the study was performed, the study type, the study period in months, the sample size, the specimen sources, the genotypic detection method, detected antimicrobial resistance genes, detected multilocus sequence types, and the year(s) of specimen collection.

### 2.3 Risk of bias assessment

The quality of each article included in the review was assessed by two independent reviewers using the National Heart, Lung, and Blood Institute (NIH) Study Quality Assessment tools adapted for each study’s design.^3^ Two tools were used: (1) the NHLBI Quality Assessment Tool for Observational Cross-Sectional Studies and (2) the NHLBI Quality Assessment Tool for Case Series Studies. The quality of each study was rated as good, fair, or poor to assess the risk of bias in the study due to flaws in study design or implementation (Supplementary file S2).

### 2.4 Statistical analysis

Due to the nature of the current systematic review, the results are presented as frequencies and percentages. Microsoft Excel was used for the quantitative analysis of the extracted data. The study period was presented as mean ± SD. The number of studies in each country and the prevalence of antimicrobial resistance genes were presented in frequencies. GraphPad Prism (version 10.2.3) (347) was used to create graphs and figures.

## 3 Results

### 3.1 Literature search and study selection

**Figure 1:**
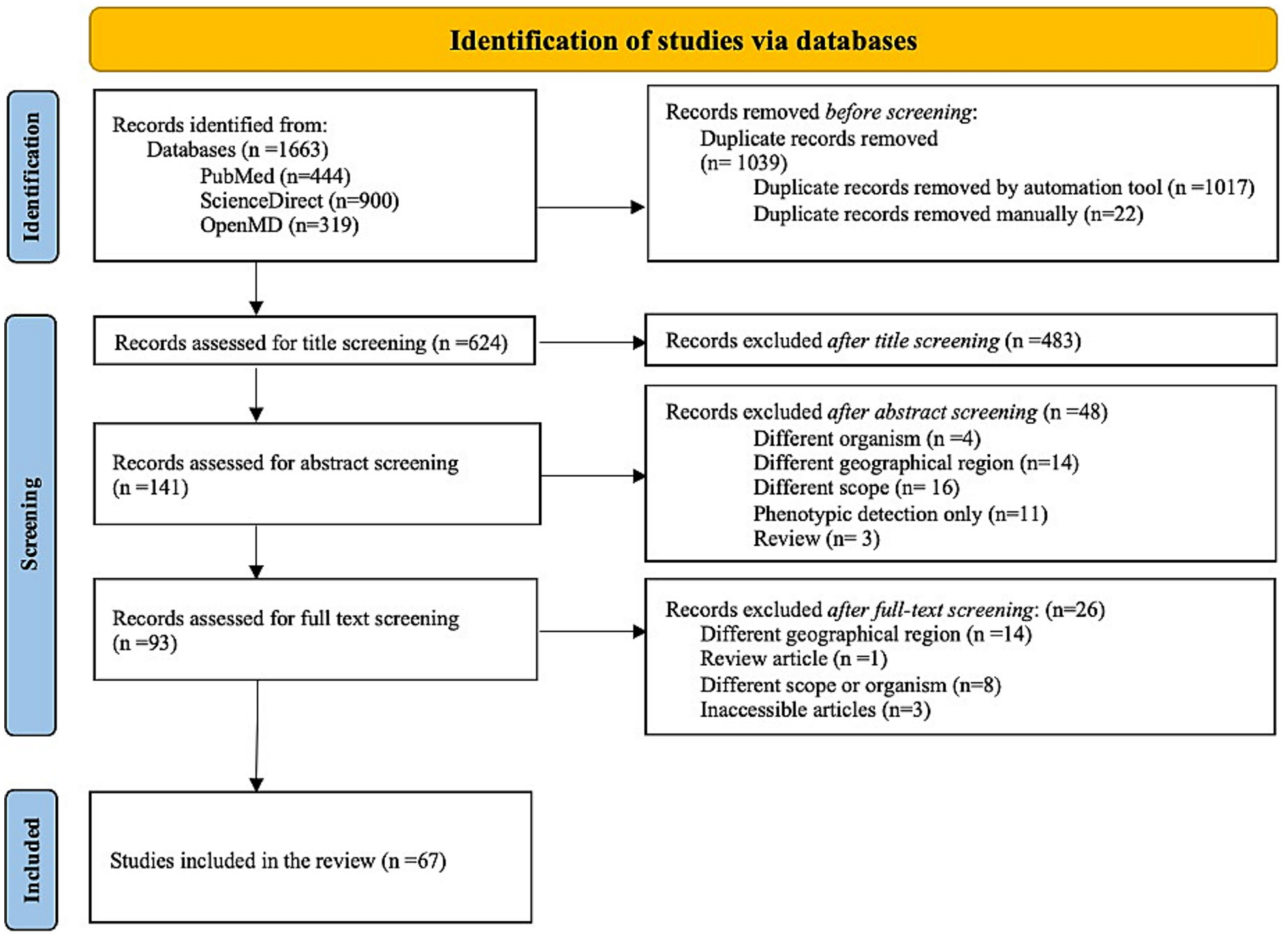
Preferred Reporting Items for Systematic Reviews and Meta-Analysis (PRISMA) flow-chart describes the results of molecular epidemiological studies of MDR *K. pneumoniae* in the GHC countries. The flow chart was downloaded from http://www.prisma-statement.org/.

A comprehensive systematic literature search was conducted to identify relevant studies on MDR of *K. pneumoniae* in the Arabian Peninsula. The search yielded a total of 1,663 studies, which were imported into the EndNote software (version 21.2). After removing duplicate studies (*n* = 1,039), the titles and abstracts of 624 studies were reviewed for possible inclusion in the current review. Out of 624 studies, 531 were excluded due to their irrelevance to the scope of the review. The remaining 93 studies were assessed for eligibility through full-text screening. Consequently, 26 articles were excluded for reasons detailed in Figure 1. Hence, a total of 67 articles met the eligibility criteria and were included in the present review.

### 3.2 Risk of bias assessment

The quality assessment results of the 67 included studies are presented in Supplementary Table S1 based on the NHLBI study quality assessment tools for observational cross-sectional studies (Supplementary Table S1A) and case series studies (Supplementary Table S1B). The NHLBI Quality Assessment Tool for Observational Cross-Sectional Studies was used for only two studies, both of which were classified as fair quality (Moghnia et al., 2021a; Sonnevend et al., 2022). Most studies were evaluated using the NHLBI Quality Assessment Tool for Case Series Studies. Out of 67 studies, 37 (55.2%) were classified as good quality, while 30 (44.7%) were classified as fair quality, as cited and described in detail in Supplementary Table S1. None of the 67 evaluated studies were classified as poor quality.

### 3.3 Overview of *Klebsiella pneumoniae* molecular epidemiology studies in the GHC countries

**Figure 2:**
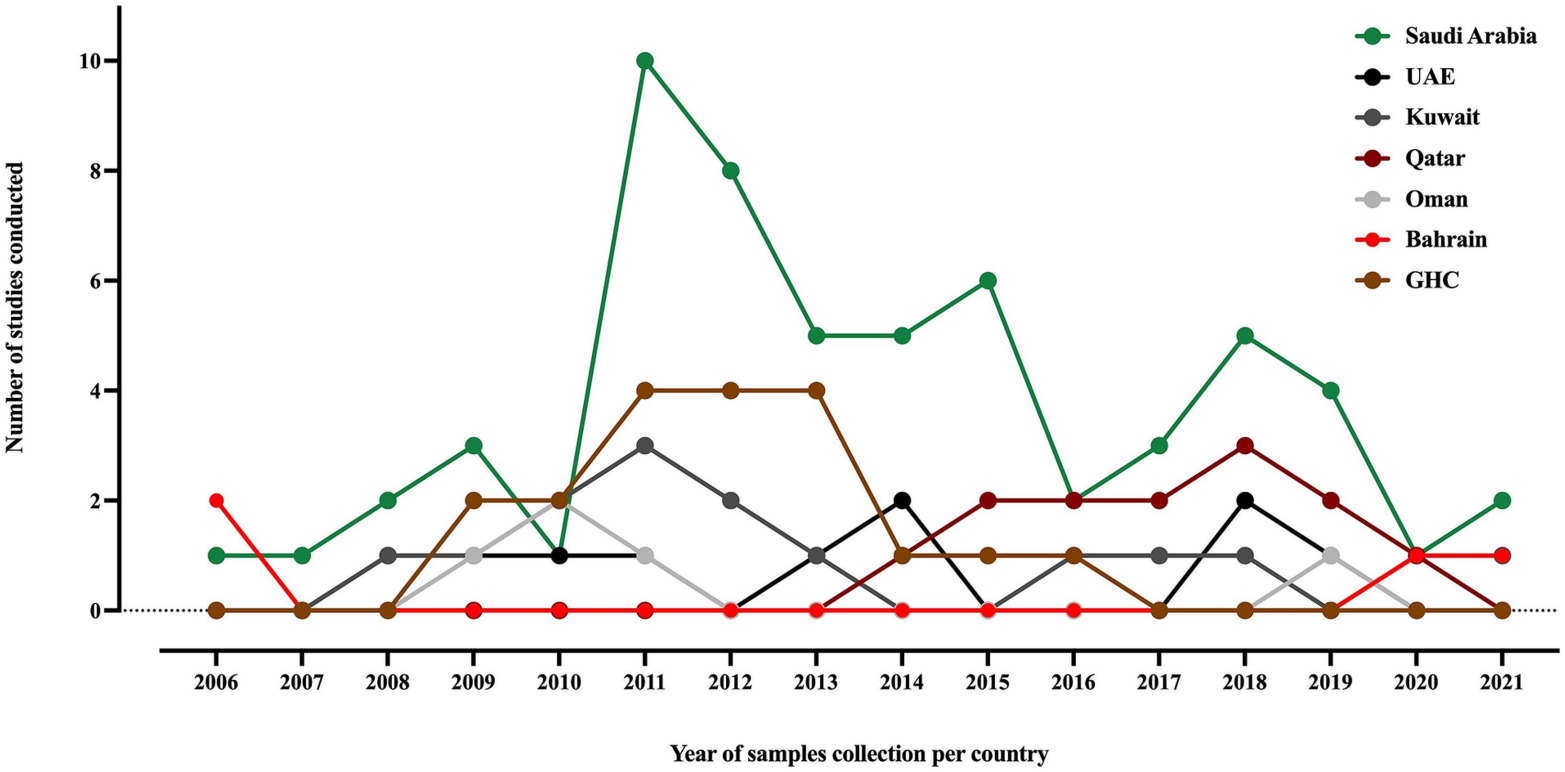
A timeline of *K. pneumoniae* studies conducted in the GHC countries, illustrating the samples collection year of each study. The label “GHC” indicates studies that involved samples collected from different GHC countries.

Out of the 67 included studies, Saudi Arabia contributed more than 60% (41/67), followed by Kuwait and the UAE, with seven and six studies, respectively. Oman and Qatar contributed four studies in each country, while Bahrain contributed only three studies. Furthermore, researchers conducted four studies using samples collected from various countries in the area (Al-Baloushi et al., 2018; Sonnevend et al., 2015; Zowawi et al., 2014; Mouftah et al., 2021). Studies of MDR *K. pneumoniae* in the GHC countries, including ESBL, carbapenemase-producing and colistin-resistant strains, were conducted over an average period of 18 ± 19 months between 2006 and 2021, as shown in Figure 2. The most extended study, which was conducted over 96 months in Saudi Arabia, was on the genetic characterization of colistin-resistant isolates (Okdah et al., 2022). Most of the studies (50.7%) focused on carbapenem-resistant *K. pneumoniae* (CRKP), followed by ESBL-producing *K. pneumoniae* (29.8%), as shown in Supplementary Table S2.

**Figure 3:**
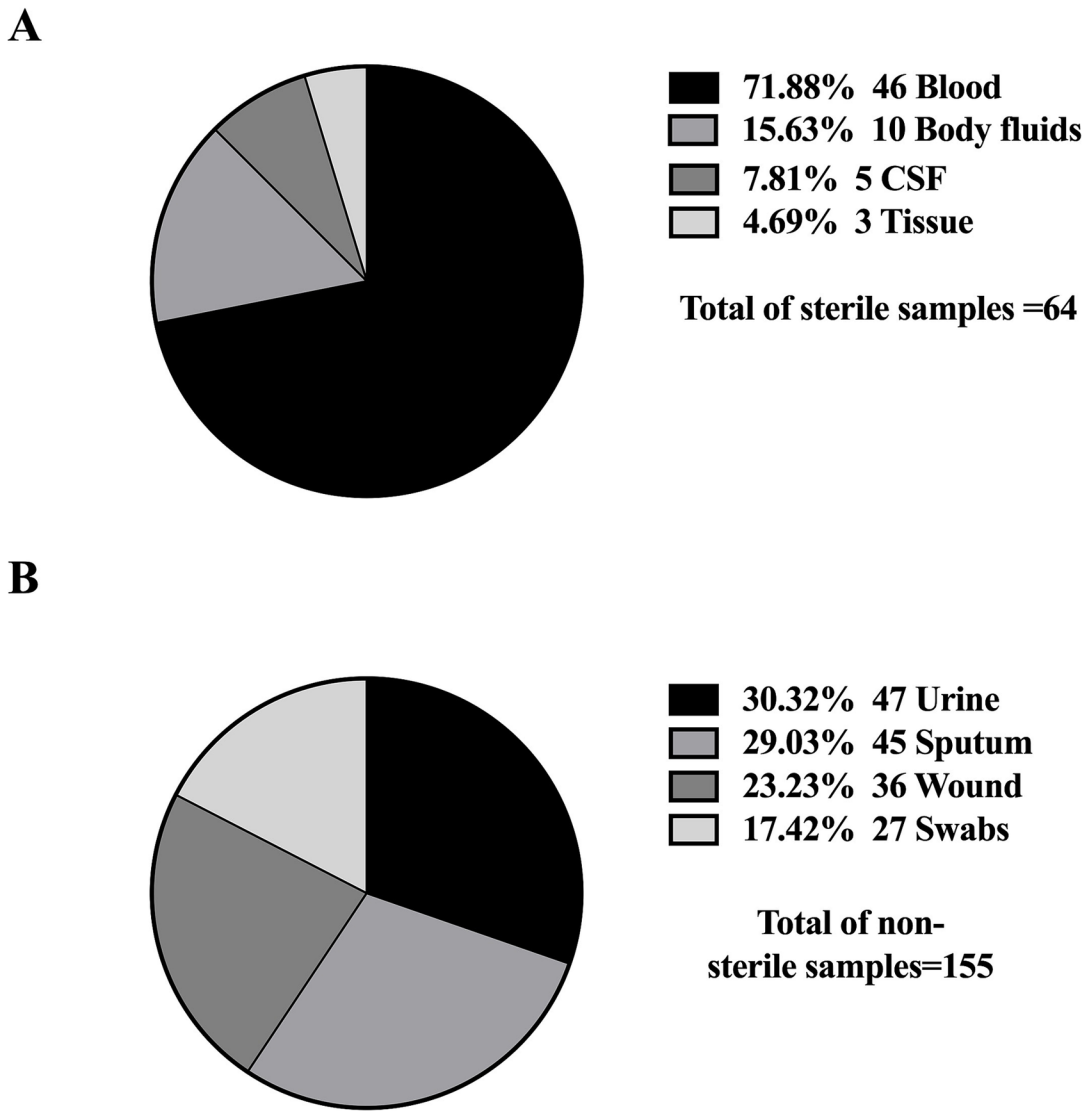
Distribution of sample types from which *K. pneumoniae* isolates were obtained. **(A)** Sterile samples: collected through invasive procedures (e.g., blood, CSF), often associated with life-threatening infections. **(B)** Non-sterile samples: obtained through non-invasive methods (e.g., urine, sputum), typically linked to less severe infections.

**Figure 4:**
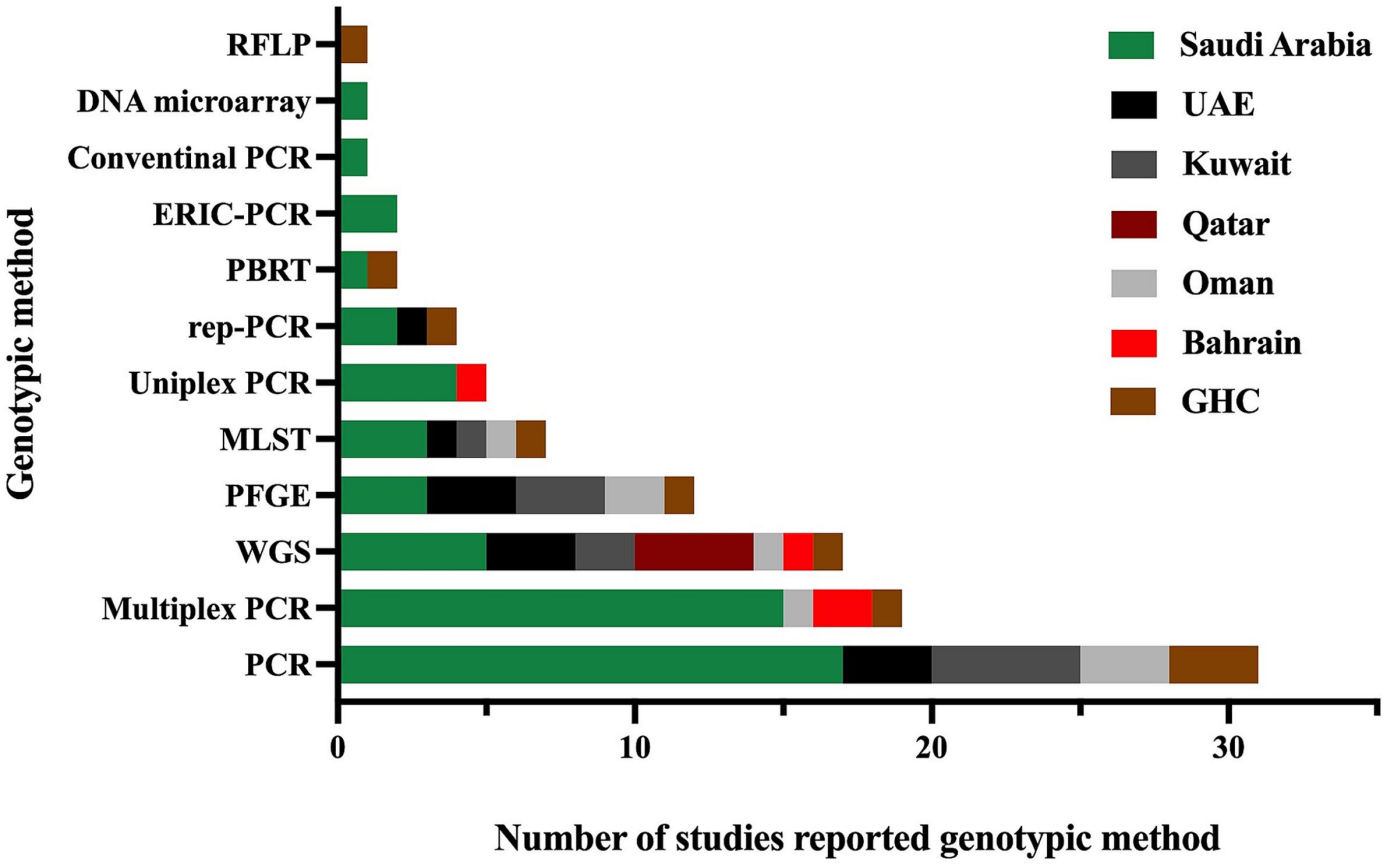
The genotypic methods used for the detection of antimicrobial resistance genes and identification of different sequence types in MDR *K. pneumoniae* were reported in studies conducted in the GHC countries. The label “GHC” reflects the studies conducted on samples obtained from different GHC countries.”

Furthermore, all studies were related to clinical settings, while only one study was conducted on livestock/animal samples (Sonnevend et al., 2022), and one study focused on collecting samples from the community (Moghnia et al., 2021a). The predominant specimen sources reported in the majority of studies were urine, sputum, and blood, as shown in Figure 3. The most commonly used genotypic method was PCR, followed by multiplex PCR and the WGS, as detailed in Figure 4.

### 3.4 Distribution and comparison of *Klebsiella pneumoniae* AMR genes in the GHC countries

An extensive analysis of the studies revealed a predominant focus on MDR *K. pneumoniae*, including ESBL, carbapenemase-producing, and colistin-resistant strains. Among the ESBL genes, *bla*_CTX-M_ was identified as the most prevalent, with two variants, *bla*_CTX-M-14_ and *bla*_CTX-M-15_, reported in most studies. The *bla*_OXA_ gene was the most identified carbapenemase gene, with *bla*_OXA-23_, *bla*_OXA-48_, *bla*_OXA-181_, and *bla*_OXA-232_ as the most common variants. However, the distribution of these predominant genes varies between the GHC countries, with certain genes reported exclusively in specific countries. This variation is likely due to the differing number of studies conducted in each country, which influences the detection and reporting of particular genes. For instance, while *bla*_CTX-M-15_ was the most predominant ESBL gene in the GHC countries, *bla*_CTX-M-14_ was reported only in Saudi Arabia, Kuwait, and Qatar. Moreover, *bla*_SHV-1_ was reported in Saudi Arabia (Tawfik et al., 2011; Uz Zaman et al., 2014; Al-Qahtani et al., 2014; Alghoribi et al., 2020) and Kuwait (Jamal et al., 2015), while *bla*_SHV-28_ was identified in the UAE (Alfaresi et al., 2011), Qatar (Eltai et al., 2020), and Oman (Poirel et al., 2011). For carbapenemase genes, *bla*_OXA_ and *bla*_NDM_ were identified in all GHC countries, with varying distributions of their variants across different countries. Conversely, *bla*_KPC_ was reported in all countries except Bahrain (Alraddadi et al., 2022; Sonnevend et al., 2022; Abid et al., 2021; Mouftah et al., 2021; Alghoribi et al., 2020; Balushi et al., 2022; Moghnia et al., 2021b; Moghnia and Al-Sweih, 2022; Azim et al., 2019; Al-Tawfiq et al., 2022; Alshahrani et al., 2022), with *bla*_KPC-2_ being the most frequently reported variant. Notably, *bla*_KPC-2_ was first reported in Saudi Arabia (Alghoribi et al., 2020). Additionally, the carbapenemase gene *bla*_IMP_ was reported only in Saudi Arabia (Alraddadi et al., 2022; Azim et al., 2019; Ejaz, 2022; Badger-Emeka et al., 2021).

Furthermore, resistance to aminoglycosides and fluroquinolones has also been observed in ESBL- or carbapenemase- producing *K. pneumoniae* in GHC countries. Among aminoglycoside-resistance genes, *aac(6′)-lb* was the most frequently identified (Al-Baloushi et al., 2018; Shibl et al., 2012; Abdalhamid et al., 2017; Al-Agamy et al., 2019; Vali et al., 2015), followed by *arm*A and *rmt*B (Sonnevend et al., 2022; Okdah et al., 2022; Alghoribi et al., 2020; Abdalhamid et al., 2017; Al Sheikh et al., 2014; Abdalhamid et al., 2017; Sonnevend et al., 2013). These genes have been documented in Saudi Arabia, the UAE, Kuwait, and Oman. However, no aminoglycoside-resistance genes were reported in Qatar and Bahrain. Among the fluoroquinolone-resistance genes, qnrB was reported in Saudi Arabia (Okdah et al., 2022; Shibl et al., 2012; Abdalhamid et al., 2017; Al-Agamy et al., 2019; Al-Agamy et al., 2018) and the UAE (Sonnevend et al., 2013), while qnrS was identified in Bahrain (Shahid et al., 2022) and Oman (Balushi et al., 2022). Interestingly, Kuwait stands out, as a study conducted there identified all three key fluoroquinolone-resistance genes: *qnr*A, *qnr*B, and *qnr*S (Vali et al., 2015). Moreover, a study conducted in Saudi Arabia identified a wide array of antimicrobial resistance genes, including *aad*A2, *ant(3′)-lh*, *arm*A, *sat*-A, *Amp*H, *aac(3′)-la*, *aph(3′)-Vib*, *str*AB, *oqx*A, *msr*(E), *cat*A, *cat*B, *sul*1, *tet* (WHO Bacterial Priority Pathogens List, 2024), *tet*(A), *tet*(D), *mcr*-1, and *dfr*A (Al-Baloushi et al., 2018; Okdah et al., 2022; Alghoribi et al., 2020; Abdalhamid et al., 2017). Additionally, other resistance mechanisms were observed, such as mutations in *gyr*A, *Par*C, *mgr*B, *Pmr*A, and *Pmr*B (Alghoribi et al., 2020; Abdalhamid et al., 2017). Structural and functional alterations in outer membrane proteins and efflux systems, including *Omp*K35, *Omp*K36, *mdt*K, *tol*C, and *acr*AB (Lagha et al., 2021), were also reported. The presence of multiple resistance mechanisms, in addition to ESBL or carbapenem resistance, classifies these isolates as MDR *K. pneumoniae*, as they demonstrate resistance to at least one antibiotic from three or more different classes. These findings underscore the multifaceted nature of antimicrobial resistance in the region and the importance of monitoring both genetic and phenotypic mechanisms to understand resistance trends comprehensively.

Alarmingly, colistin-resistant *K. pneumoniae* has been documented in three studies conducted in GHC countries. Chromosome-mediated colistin resistance, driven by *mgr*B mutations, was identified in two studies from Saudi Arabia (Okdah et al., 2022; Zaman et al., 2018). Additionally, plasmid-mediated colistin resistance associated with the *mcr-*1 gene was reported in two studies, one from Saudi Arabia (Okdah et al., 2022) and the other from the UAE (Sonnevend et al., 2022).

**Figure 5:**
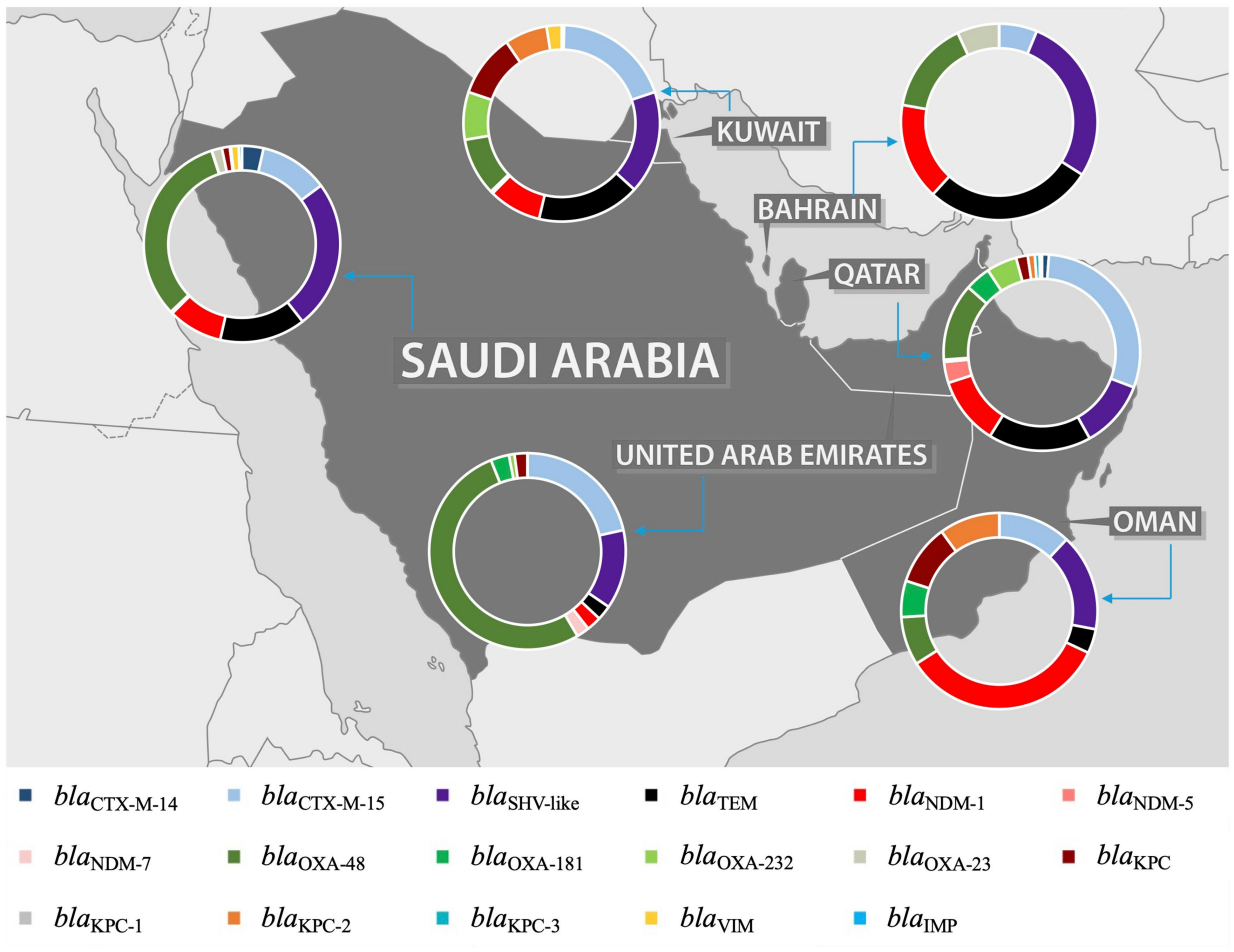
Distribution of most prevalent ESBL and carbapenemase genes in the GHC Countries. The map figure was licensed from Shutterstock (https://www.shutterstock.com) and modified using PowerPoint to express the prevalence of ARGs in each country of GHC.

**Figure 6:**
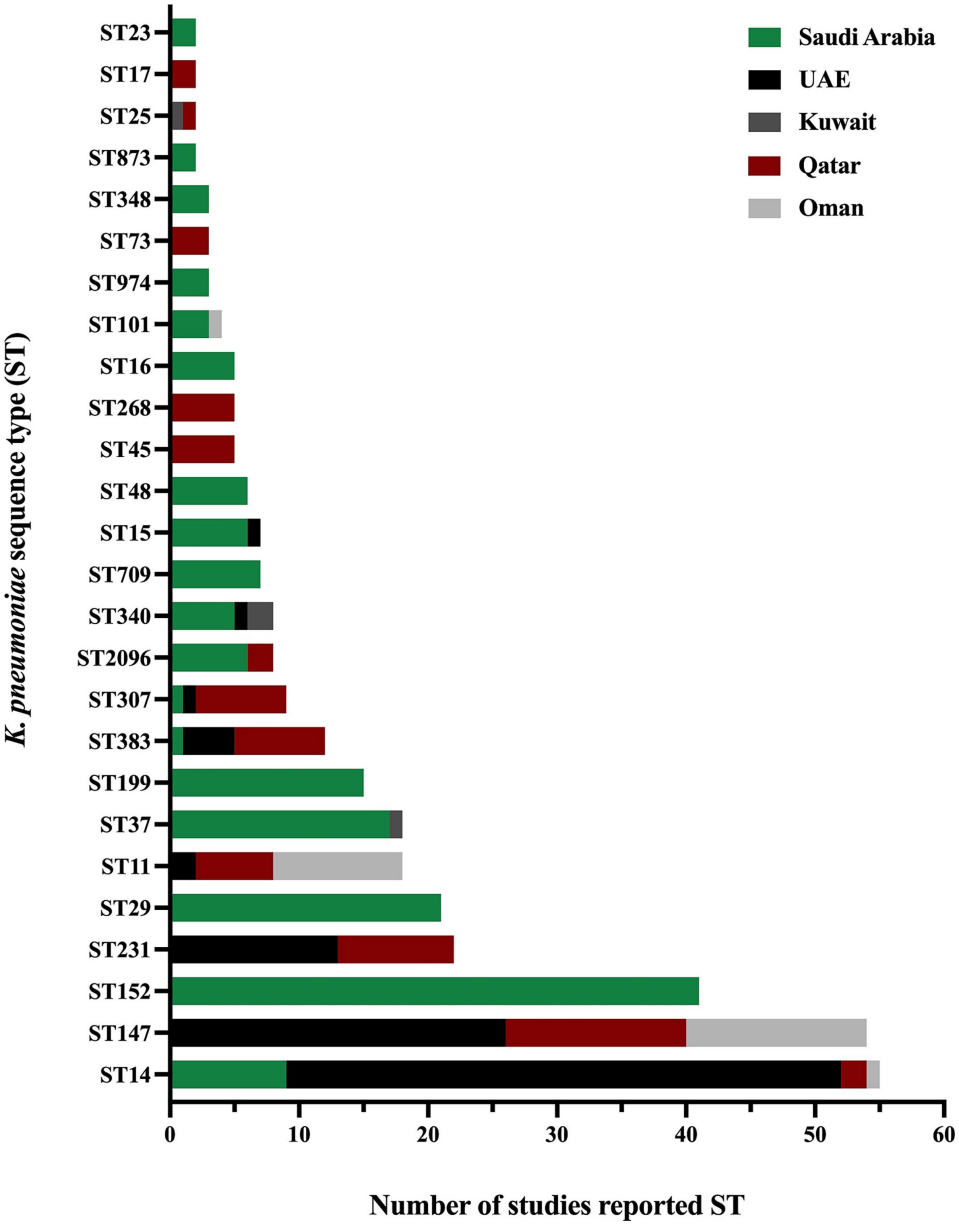
The distribution of *K. pneumoniae* sequence types in the GHC countries shows the variety of *K. pneumoniae* sequence types in different geographical regions.

The presence and absence of various antimicrobial resistance genes across different GHC countries are shown in Figure 5. Even though many studies did not include the analysis and identification of *K. pneumoniae* sequence types, some studies reported the presence of specific *K. pneumoniae* sequence types among their isolates. As some sequence types were reported in different geographical regions, such as ST11, ST14, ST15, ST37, ST101, ST147, ST307, ST340, ST383, and ST2096, other sequence types were specific to some regions, among others, as illustrated in (Figure 6).

### 3.5 *Klebsiella pneumoniae* AMR genes in the Kingdom of Saudi Arabia

The Kingdom of Saudi Arabia contributed the majority of the data from the GHC countries, with a total of 41 studies (61.1% of the total) included in this review conducted between 2010 and 2022. Most studies utilized multiplex PCR to detect ESBL and carbapenem-resistance genes, while a subset of studies incorporated WGS in their methodology. The studies demonstrated that the ESBL gene (*bla*_CTX-M_) was the most predominant resistance gene over the years, followed by β-lactamase genes (*bla*_TEM_ and *bla*_SHV_)_,_ regardless of the variants. Additionally, various carbapenemase genes were detected in Saudi Arabia, including *bla*_OXA-48_, *bla*_NDM-1_, *bla*_KPC-2_, *bla*_VIM_, and *bla*_IMP_. However, *bla*_OXA-48_ and *bla*_NDM-1_ emerged as the most prevalent genes over the years. In 2013 (Shibl et al., 2013), A. Shibl and his team reported *bla*_OXA-48_ and *bla*_NDM-1_ for the first time in Saudi Arabia, while *bla*_KPC_ was reported for the first time in 2019 by N. Azim and collaborators (Azim et al., 2019). In 2019, M. Khan reported the presence of triple co-producing carbapenemase genes (*bla*_OXA-48_, *bla*_NDM-1_, and *bla*_KPC_) in 80% of isolates collected from clinical samples using PCR methods (Khan et al., 2019). However, these results have not been subsequently confirmed, indicating the need for further investigation. In contrast, the triple co-producing carbapenemase genes (*bla*_OXA-48-like_, *bla*_NDM-1_, and *bla*_KPC_) were identified in 1.4% of isolates in another study by A. Alshahrani and others in 2022 (Alshahrani et al., 2022). A combination of three carbapenemase genes (*bla*_OXA-48_, *bla*_NDM-1_, and *bla*_VIM_) was reported in 21.7% of clinical isolates, while a combination of four carbapenemase genes (*bla*_OXA-23_, *bla*_OXA-48_, *bla*_NDM-1_, and *bla*_VIM_) was reported in a single clinical isolate in 2022 by R. Booq and the team (Booq et al., 2022). The emergence of ESBL and carbapenemase genes has been well-documented in Saudi Arabia. However, the use of colistin as a last-line treatment has led to the alarming emergence of colistin resistance in clinical isolates of *K. pneumoniae*. Multiple studies have reported colistin-resistant *K. pneumoniae* in clinical settings, with chromosomally mediated resistance primarily due to mutations in the *mgr*B, *pmr*A, *pmr*B, and *pho*Q genes. Furthermore, plasmid-mediated colistin resistance has been identified by acquiring the *mcr-1* gene (Okdah et al., 2022; Zaman et al., 2018). The molecular typing results of *K. pneumoniae* isolates revealed that the most predominant clonal group is clonal complex 14 (CC14), which includes the sequence types ST14 and ST2096 in a study conducted in 2020 and 2022 (Okdah et al., 2022; Khdary et al., 2020). Several studies have analyzed the MLST of *K. pneumoniae* isolates, identifying various sequence types. Additionally, other sequence types reported in the literature include ST11, ST23, ST37, ST147, ST307, ST340, and ST383 (Al-Baloushi et al., 2018; Sonnevend et al., 2015; Okdah et al., 2022; Uz Zaman et al., 2014; Alghoribi et al., 2020; Al-Agamy et al., 2019; Zaman et al., 2018; Moglad et al., 2022; Uz Zaman et al., 2018; Almogbel et al., 2021). The predominance of CC14 highlights its significance in the epidemiology of *K. pneumoniae* in Saudi Arabia. Identifying these sequence types underscores the genetic diversity of *K. pneumoniae* strains circulating in clinical settings, contributing to the complexity of managing infections caused by this pathogen.

### 3.6 *Klebsiella pneumoniae* AMR genes in the United Arab Emirates

The United Arab Emirates (UAE) contributed the third-highest number of studies, following Saudi Arabia and Kuwait, with a total of six studies out of 67 (8.9%). Most of these studies focused on investigating AMR on clinical samples (Sonnevend et al., 2022; Alfaresi et al., 2011; Sonnevend et al., 2013; Sonnevend et al., 2017; Zowawi et al., 2015), with only one study conducted on livestock origin samples (Sonnevend et al., 2022). The majority reported ESBL-producing *K. pneumoniae* or CRKP, with one study highlighting the presence of colistin-resistant *K. pneumoniae* in a sample of livestock origin (Sonnevend et al., 2022). In addition, most clinical samples were recovered from various infection sites, including urine, blood, respiratory, and wound swabs. At the same time, the livestock origin specimens are comprised of fecal samples collected from birds in poultry farms. Various resistance genes were reported in the UAE, including β-lactamase genes *bla*_TEM-1_ (Sonnevend et al., 2017) and *bla*_SHV-11_ (Sonnevend et al., 2013) and ESBL genes *bla*_SHV-12_ (Sonnevend et al., 2022; Sonnevend et al., 2013), *bla*_SHV-28_ (Alfaresi et al., 2011), *bla*_SHV-36_ (Zowawi et al., 2015), and *bla*_CTX-M-15_ (Sonnevend et al., 2022; Alfaresi et al., 2011; Sonnevend et al., 2013; Sonnevend et al., 2017; Zowawi et al., 2015), with *bla*_CTX-M-15_ identified as the most predominant ESBL gene in the UAE. Carbapenemase genes were reported in four studies, including *bla*_OXA-48_ (Sonnevend et al., 2022), *bla*_OXA-181_ (Sonnevend et al., 2017; Zowawi et al., 2015), *bla*_NDM-1_ (Sonnevend et al., 2022; Sonnevend et al., 2013), *bla*_NDM-5_ (Sonnevend et al., 2017), and *bla*_KPC_ (Sonnevend et al., 2022). Notably, colistin-resistant *K. pneumoniae* was reported in several studies within the time frame of this literature review. Chromosomal mutations were detected in clinical samples, specifically in the genes *mgrB*, *phoP*, *phoQ*, *pmrA*, and *pmrB* (Sonnevend et al., 2022; Sonnevend et al., 2017). The *mcr-1* gene, on the other hand, was identified in fecal samples collected from birds in poultry farms (Sonnevend et al., 2022). However, no *mcr* genes were found in the colistin-resistant *K. pneumoniae* strains subjected to WGS from clinical samples. MLST analysis in the UAE indicated ST14 was the most predominant sequence type, followed by ST147 and ST231 (Sonnevend et al., 2022; Al-Baloushi et al., 2018; Sonnevend et al., 2015; Sonnevend et al., 2013; Sonnevend et al., 2017; Zowawi et al., 2015). Additional studies reported the detection of various sequence types in the UAE, including ST11, ST231, ST307, ST340, ST383, and ST1318 (Sonnevend et al., 2022; Sonnevend et al., 2022; Al-Baloushi et al., 2018; Sonnevend et al., 2015; Sonnevend et al., 2013).

### 3.7 *Klebsiella pneumoniae* AMR genes in Kuwait

Kuwait contributed the second-highest number of studies, following Saudi Arabia, with a total of seven studies reporting the ESBL producing *K. pneumoniae* (Vali et al., 2015; Al Sweih et al., 2011; Dashti et al., 2010), or CRKP (Jamal et al., 2015; Moghnia et al., 2021b; Moghnia and Al-Sweih, 2022; Jamal et al., 2013). Most studies were conducted on clinical samples, with only one study focusing on healthy food handlers from community settings (Moghnia et al., 2021b). In 2008, a β-lactamase gene (*bla*_TEM-1_) and an ESBL gene (*bla*_CTX-M-15_) were identified in all 14 isolates of ESBL-producing *K. pneumoniae* over a period of 2 months (Al Sweih et al., 2011). Genotypic studies from Kuwait revealed *bla*_TEM-1_ and *bla*_SHV-11_ as predominant β-lactamases genes, while *bla*_CTX-M-15_ was identified as the predominant ESBL genes in ESBL-producing *K. pneumoniae.* Interestingly, one study showed an outbreak reporting a number of isolates that were phenotypically resistant to cephalosporin antibiotics. Molecular genetics analysis showed that these isolates harbor *bla*_SHV-112_ gene, which is classified as an ESBL genes (Dashti et al., 2010). Moreover, several studies have reported the major carbapenemase genes, which include *bla*_OXA-48_, *bla*_OXA-181_, *bla*_OXA-232,_ *bla*_NDM-1_, and *bla*_KPC-2_ (Jamal et al., 2015; Moghnia et al., 2021b; Moghnia and Al-Sweih, 2022; Jamal et al., 2013). Notably, other *bla*_KPC_ variants, including *bla*_KPC-18_ and *bla*_KPC-29_, were first identified in Kuwait from community samples between 2016 and 2018 (Moghnia et al., 2021a). The detailed list of all ESBL and carbapenemase genes is presented in Supplementary Table S2. Results of MLST analysis of *K. pneumoniae* isolates obtained from food handlers reported various sequence types composed of ST10, ST38, ST295, ST1415, and ST1876 (Moghnia et al., 2021b). In addition, three new CRKP sequence types (ST1592, ST1593, and ST1594) were reported in a study conducted between 2011 and 2013 on clinical samples (Jamal et al., 2015). Other sequence types were identified in different studies including ST16, ST25, ST37, ST107, ST485, ST677, ST3495, and ST4743 (Jamal et al., 2015; Moghnia and Al-Sweih, 2022). However, all identified STs in Kuwait were reported as singleton with no observed predominance.

### 3.8 *Klebsiella pneumoniae* AMR genes in Qatar

Four studies were conducted in Qatar to detect ESBL and carbapenemase genes in *K. pneumoniae* isolates obtained from clinical samples such as urine, blood, respiratory, and wound swabs (Abid et al., 2021; Eltai et al., 2020; Perez-Lopez et al., 2020; Pérez-López et al., 2021). These major resistance genes were the most predominant genes in all studies conducted in Qatar between 2015 and 2019 (Eltai et al., 2020; Perez-Lopez et al., 2020; Pérez-López et al., 2021). A study conducted from 2014 to 2017 reported the presence of *bla*_OXA-48_, *bla*_OXA-181_, *bla*_OXA-232_, *bla*_NDM-1_, *bla*_NDM-5_, *bla*_NDM-7_, *bla*_KPC-2_, *bla*_KPC-3_ and *bla*_VIM-2_. In virous sequence types, including ST11, ST147, ST231 and ST383 of which ST147 being the most predominant (Abid et al., 2021). Additionally, co-occurrence of resistance genes was observed, including combinations such as (*bla*_OXA-48_ and *bla*_NDM-5_), (*bla*_OXA-181_ and *bla*_NDM-1_), and (*bla*_OXA-181_ and *bla*_NDM-5_) and (*bla*_NDM-1_ and *bla*_KPC-3_) (Abid et al., 2021). Other studies confirmed the presence of *bla*_OXA-48_, *bla*_NDM-1_, and *bla*_NDM-5_ as the most widely disseminated carbapenemase genes in Qatar (Eltai et al., 2020; Perez-Lopez et al., 2020; Pérez-López et al., 2021). The analysis highlighted *bla*_OXA-48_ and *bla*_NDM-1_ as the dominant carbapenemase genes identified in the country. The molecular typing of *K. pneumoniae* isolates resulted in the identification of different sequence types through several studies over the years. For instance, a study between 2015 and 2019 identified the following sequence types: ST11, ST25, ST147, ST231, ST383, ST716, ST792, and ST2096 (Eltai et al., 2020), while a study in 2018 reported the presence of ST45, ST268 and ST307 *K. pneumoniae* sequence types (Perez-Lopez et al., 2020). Other sequence types were identified between 2018 and 2020, composed of ST14, ST17, and ST73 (Pérez-López et al., 2021).

### 3.9 *Klebsiella pneumoniae* AMR genes in Sultanate of Oman

Four studies in Oman were eligible to be included in the current systematic review (Poirel et al., 2011; Balushi et al., 2022; Potron et al., 2011; Dortet et al., 2012). These studies employed molecular methods to investigate the presence of antimicrobial resistance genes in *K. pneumoniae,* primarily using PCR as the genotypic method. However, only one study incorporated WGS to further elucidate these characteristics (Balushi et al., 2022)^.^ These studies were conducted in 2011, 2012, and 2022 focused on either *K. pneumoniae* or CRKP. Studies have reported resistance mechanisms associated with the β-lactamase gene (*bla*_SHV_) and ESBL gene (*bla*_CTX-M_), which were identified as the predominant resistance mechanisms. These studies also identified significant carbapenemase genes, including *bla*_NDM-1_ in 2011, 2012, and 2022 (Poirel et al., 2011; Balushi et al., 2022; Dortet et al., 2012), and *bla*_KPC-2_ in 2022 (Balushi et al., 2022). Additionally, the co-occurrence carbapenemase genes, specifically *bla*_OXA-181_ and *bla*_NDM-1_, were observed in 2012 by L. Dortet and colleagues (Dortet et al., 2012). Furthermore, MLST analysis across all studies in Oman identified ST11 and ST147 as the major sequence types (Al-Baloushi et al., 2018; Sonnevend et al., 2015; Balushi et al., 2022; Potron et al., 2011; Dortet et al., 2012). These sequence types were the most predominant in the region, highlighting their significant role in the epidemiology of *K. pneumoniae*. Other sequence types reported as singletons include ST14, ST15, ST101, ST340, ST372, ST753, and ST754 (Poirel et al., 2011; Balushi et al., 2022).

### 3.10 *Klebsiella pneumoniae* AMR genes in the Kingdom of Bahrain

Three studies were conducted in Bahrain to evaluate the presence of antimicrobial resistance genes in *K. pneumoniae* obtained from clinical samples, with notable time gaps between these three studies (Shahid et al., 2022; Bindayna and Murtadha, 2011; Shahid et al., 2014). The first study investigated the prevalence of ESBL-producing *K. pneumoniae* over 6 months. This study analyzed clinical samples, including urine, blood, respiratory, and wound swabs, and found that nearly all samples tested positive for ESBL. The most prevalent ESBL gene identified was *bla*_CTX-M_ (90%), followed by β-lactamase genes *bla*_TEM_ and *bla*_SHV_ (80%). An important finding was the co-occurrence of these genes, with 70% of isolates harboring all three (*bla*_TEM_, *bla*_SHV_ and *bla*_CTX-M_) and only 10% containing just *bla*_SHV_ and *bla*_CTX-M_ (Bindayna and Murtadha, 2011). The second study reported the prevalence of ESBL-producing *K. pneumoniae* over 7 months, analyzing clinical samples that included urine, blood, respiratory, and wound swabs. All the *K. pneumoniae* samples in this study tested positive for the presence of the *bla*_CTX-M-15_ gene (Shahid et al., 2014). The third study, conducted over 7 months, focused on detecting CRKP in clinical samples, with blood being the most common sample type, followed by urine and respiratory. The study identified multiple carbapenemase genes, including *bla*_OXA-23_, *bla*_OXA-48_, *bla*_OXA-51_ and *bla*_NDM-1_. Additionally, some isolates co-produced multiple carbapenemase genes, revealing double or triple carbapenemase gene combinations, including *bla*_OXA-48_, *bla*_OXA-51_ and *bla*_NDM-1_. Notably, 100% of the isolates in this study harbored the *qnrS* gene alongside multiple carbapenemase genes, contributing to the multidrug-resistant profile (Shahid et al., 2022).

## 4 Discussion

This systematic review underscores the complex and multifaceted burden posed by MDR *K. pneumoniae* in the GHC countries. The majority of the studies included focused on investigating MDR *K. pneumoniae* on clinical samples, with limited exploration of its presence in community and environmental settings, such as food handlers (Moghnia et al., 2021b) and poultry farms (Sonnevend et al., 2022). Hospital-based studies partially reflect community health, but the One Health approach and molecular epidemiology are needed to better track and manage MDR *K. pneumoniae* across human, animal, and environmental interfaces. This significant gap in the data limits our understanding of the broader epidemiological landscape and potential non-clinical reservoirs of MDR *K. pneumoniae*, which are critical for comprehensive public health strategies.

The Kingdom of Saudi Arabia contributed the majority of research within the GHC region, followed by Kuwait and the United Arab Emirates. However, the variability in study designs, detection methods, sample sizes, and targeted genes across these studies complicates direct comparisons. It limits the potential for meta-analysis, thereby underscoring the need for standardized methodologies in future research. Despite these methodological challenges, the application of NGS in certain studies has yielded valuable insights into the molecular epidemiology of MDR *K. pneumoniae*, facilitating the identification and tracking of prevalent resistance genes within the region.

The widespread prevalence of resistance genes, notably the β-lactamase gene *bla*_TEM_ and the ESBL gene *bla*_CTX-M-15,_ emerged as a critical finding across the GHC countries. Our analysis of the prevalence of the *bla*_SHV_ gene revealed regional variations. The β-lactamase gene (*bla*_SHV-1_) was exclusively found in Kuwait and Saudi Arabia. The β-lactamase gene (*bla*_SHV-11_) and ESBL genes (*bla*_SHV-12_) were identified in Saudi Arabia, UAE, and Kuwait, while the ESBL genes (*bla*_SHV-28_ and *bla*_SHV-38_) were detected in the UAE, Qatar, and Oman. This observed geographic variability in the prevalence of *bla*_SHV_ suggests regional differences in disseminating these resistance mechanisms, which may reflect local healthcare practices or environmental factors. The plasmid-mediated dissemination of ESBL genes has significantly compromised the efficacy of critical antimicrobials, including penicillins and other ß-lactams, across the GHC countries, mirroring global trends in the spread of antimicrobial resistance (Paterson et al., 2003; Saravanan et al., 2018; Agyepong et al., 2019; Manandhar et al., 2020; Nordmann and Mammeri, 2007). These findings align with international trends, such as those reported in clinical settings in Africa, where high prevalence rates of *bla*_TEM_, *bla*_SHV_, and *bla*_CTX-M_ have been documented, further underscoring the pervasive nature of these resistance genes.

The first identification of *bla*_CTX-M-15_ occurred in Greece during an ICU infection outbreak (Poulou et al., 2013), spreading rapidly to other countries, including Spain, France, Italy, Poland, Tunisia and Japan, underscoring its global significance (Marcade et al., 2013; Elhani et al., 2010; Robin et al., 2017; Rodrigues et al., 2014; Ochońska et al., 2021; Mrowiec et al., 2019; de Walthoffen et al., 2011). This gene, along with *bla*_TEM_ and *bla*_SHV_, has become widely disseminated across regions such as Portugal, North America, Argentina, Australia, South Africa, Turkey, and the United States (Paterson et al., 2003; Rodrigues et al., 2014). These findings align with international trends observed in clinical settings, including those in Africa, where high prevalence rates of *bla*_TEM_, *bla*_SHV_, and *bla*_CTX-M_ have been documented (Saravanan et al., 2018; Agyepong et al., 2019). The coexistence of these genes on a global scale mirrors the patterns observed in the GHC countries, suggesting that international travel and migration may contribute to the genetic diversity and spread of β-lactamase and ESBL genes in this region.

The widespread dissemination of ESBL-producing *K. pneumoniae* has necessitated the increased use of carbapenems as a last-resort treatment option [Infectious Diseases Society of America (IDSA), 2011]. Consequently, this reliance has led to the global emergence of CRKP, posing a significant public health threat (Tamma and Simner, 2018). Over 50% of the studies in the GHC region focused on CRKP, highlighting the regional concern over this growing resistance. The initial identification of *bla*_OXA-48_ and *bla*_NDM-1_ carbapenemase genes marked the beginning of CRKP’s emergence, which has since been reported in nosocomial outbreaks worldwide, including in the GHC countries.

Notably, *bla*_OXA-48_ has been the most frequently reported carbapenemase gene in the region, although specific *bla*_OXA_ variants vary by geography. This aligns with findings from Tehran, where *bla*_OXA-48_ was prevalent among CRKP isolates (Solgi et al., 2020). In contrast, studies from Nepal identified blaNDM-2 as the predominant carbapenemase gene in CRKP (Manandhar et al., 2020; Pyakurel et al., 2021). Within the GHC countries, *bla*_NDM-1_ emerged as the second most common carbapenemase gene, with other variants like *bla*_NDM-5_ and *bla*_NDM-7_ being reported in Saudi Arabia, Qatar, and the UAE (Abid et al., 2021; Ejaz, 2022; Sonnevend et al., 2017).

Carbapenemase gene *bla*_NDM-1_ is particularly notable as it is the most widely distributed metallo-β-lactamase enzyme in Enterobacteriaceae across South and Southeast Asia (Hsu et al., 2017). The coexistence of multiple carbapenemase genes is a significant concern, with plasmids carrying *bla*_NDM_ are frequently linked to the presence of both *bla*_OXA-48_ and *bla*_VIM_ genes (Nordmann and Mammeri, 2007). Similar co-occurrence patterns, the combination of more than one carbapenemase gene, such as *bla*_OXA-181_ and *bla*_NDM-1_ or *bla*_NDM-5,_ was reported among hospitalized patients in India (Castanheira et al., 2011) and Singapore (Balm et al., 2013). Additionally, *bla*_OXA-232_ was identified in the GHC countries in agreement with a previous report in South India (Shankar et al., 2019).

Recently, *bla*_KPC_ was reported in different regions in the GHC countries, with *bla*_KPC-2_ being the most reported variant. The first identification of *bla*_KPC-2_ was reported in New York in 2004 (Bratu et al., 2005). Since 2004, *bla*_KPC-2_ was reported in different geographical regions globally, such as France, Columbia and China (Queenan and Bush, 2007). A combination of *bla*_KPC_ gene and other carbapenemase genes, including *bla*_OXA181_ and *bla*_NDM-1,_ was also reported (Moghnia et al., 2021b). The carbapenemase gene *bla*_IMP_ was only reported in Saudi Arabia. Differences in the prevalence of carbapenemase genes might be due to variations in international travel and the way these genes spread within individual countries in the GHC countries. Alarmingly, the increase in CRKP prevalence worldwide has resulted in increased use of colistin with the consequence of emerging resistance (Gelband et al., 2015).

Colistin is often considered the last resort for treating infections caused by MDR gram-negative bacteria such as CRKP. However, the recent emergence and dissemination of the colistin resistance gene *mcr*-1 brought significant challenges to public health. Alarmingly, the colistin-resistant gene *mcr*-1 was identified and reported in two different countries in the GHC countries: Saudi Arabia (Okdah et al., 2022) and the UAE (Sonnevend et al., 2022) in 2022. The UAE study identified a surprising variety of mcr-producing colistin-resistant strains environmentally in the fecal specimens of broiler poultry. These findings match the data reported from neighboring countries, such as Lebanon and Egypt (Al-Mir et al., 2021; Sadek et al., 2021). A study in Saudi Arabia found colistin-resistant strains of *K. pneumoniae* in hospitals (Okdah et al., 2022). This comes alongside worldwide studies, such as Egypt, North India, South America, North America, Africa, and China, that reported similar antibiotic-resistant bacteria carrying a specific gene (*mcr-1*) on a plasmid (Singh et al., 2021; Liu et al., 2021; Attalla et al., 2023; Zurfuh et al., 2016; Arcilla et al., 2016).

Furthermore, a study in China linked the overuse of colistin in animal feed to the emergence of a colistin-resistant population. Other studies suggested that the plasmid-mediated *mcr-1* gene can quickly spread from the environment and animals into key human epidemic strains (Zurfuh et al., 2016; Liu et al., 2016; Grami et al., 2016; Elmonir et al., 2021). Therefore, the Food and Agriculture Organization of the United Nations and the Codex Alimentarius Commission established the risk management options that all countries should adopt to control the spread of antimicrobial resistance in agriculture (Canton, 2021; Joint, 2015). Beyond the previously mentioned plasmid-borne mechanism, *mgrB*, *phoP*, *phoQ*, *pmrA*, and *pmrB* mutations have also been identified as a colistin-resistance mechanism in Saudi Arabia (Okdah et al., 2022; Zaman et al., 2018) United Arabic Emirate (Sonnevend et al., 2022; Sonnevend et al., 2017).

A study conducted using community-derived samples obtained from food handlers in Kuwait identified the presence of different carbapenemase genes with various variants such as *bla*_OXA_ (*bla*_OXA-48_, *bla*_OXA-181_, and *bla*_OXA-232_), *bla*_NDM_ (*bla*_NDM-1_, *bla*_NDM-6_, and *bla*_NDM-7_), and *bla*_KPC_ (*bla*_KPC-2_, *bla*_KPC-18_ and *bla*_KPC-29_) (Moghnia et al., 2021b). This is in agreement with Castanheira et al. (2011) and Rojas et al. (2017) studies that reported the presence of *bla*_OXA-181_ among *K. pneumoniae* colonizing the gastrointestinal tract of patients admitted to the hospital in the Indian subcontinent (Castanheira et al., 2011; Rojas et al., 2017). The spread of these genes in Kuwait might be explained by the high rate of Indian food handlers working in the region. On the other hand, a study in Germany reported the identification of different genes, such as *bla*_SHV-27_, *bla*_SHV-41_, *bla*_CTX-M_, *bla*_OXY_, *bla*_OKP_, *bla*_LEN_, and *mcr-1* from animals and food products (Klaper et al., 2021).

Implementing robust AMR surveillance programs at both national and regional levels within the GHC is crucial for understanding and mitigating the burden of MDR *K. pneumoniae.* Such programs are essential for providing timely and accurate data on the prevalence and distribution of resistance genes, which is critical for informing public health strategies and treatment guidelines. A well-coordinated AMR surveillance network across the GHC countries would enable the detection of emerging resistance trends, facilitate data sharing between nations, and support the development of targeted interventions. Without these efforts, the GHC region faces the risk of being unprepared for the rapid spread of MDR *K. pneumoniae*, potentially leading to increased healthcare costs, higher morbidity and mortality rates, and diminished effectiveness of available antibiotics. A comprehensive AMR surveillance program is vital for addressing current threats and safeguarding future public health in the region.

## 5 Conclusion

Limiting our search to English-language articles retrieved from major electronic databases allowed us to identify research published in high-impact journals. However, accurately determining the true prevalence of MDR *K. pneumoniae* in the region proved challenging due to the heterogeneity observed in the included studies. Due to these limitations, the findings of this systematic review are not exclusive, but they highlighted the genetic basis of increased resistance in *K. pneumoniae* in the area. Limited environmental and One Health studies in the GHC countries leave a gap in estimating the dissemination mechanism of MDR *K. pneumoniae* from the environment or food into human pathogens. The current review found a high prevalence of β-lactamase and ESBL-producing *K. pneumoniae* carrying *bla*_TEM_, *bla*_SHV_, and *bla*_CTX-M_ genes, with significant variation between countries.

Moreover, an increased spread of CRKP was reported and found to pose a serious public health risk in the GHC countries. It is important to note that combined efforts to combat the problem of rapid dissemination of MDR *K. pneumoniae* in the region. In addition, a multi-disciplinary approach is needed to better understand the emergence and dissemination of MDR *K. pneumoniae* in the region as the one health strategy aims to reduce the rise and spread of AMR at the human-animal-environment interface across the globe.

## Data Availability

The original contributions presented in the study are included in the article/Supplementary material, further inquiries can be directed to the corresponding author.

## Footnotes

^1^accessed from https://www.prisma-statement.org.

^2^ https://mcw.libguides.com/EBM/PICO

^3^accessed from https://www.nhlbi.nih.gov/health-topics/study-quality-assessment-tools.
